# Methylated Host Cell Gene Promoters and Human Papillomavirus Type 16 and 18 Predicting Cervical Lesions and Cancer

**DOI:** 10.1371/journal.pone.0129452

**Published:** 2015-06-09

**Authors:** Nina Milutin Gašperov, Ivan Sabol, Pavao Planinić, Goran Grubišić, Ivan Fistonić, Ante Ćorušić, Magdalena Grce

**Affiliations:** 1 Rudjer Boskovic Institute, Division of Molecular Medicine, Zagreb, Croatia; 2 Department of Gynecologic Oncology, Clinic for Gynecology and Obstetrics, Clinical Hospital Center Zagreb, Zagreb, Croatia; 3 Obstetrics and Gynaecology Clinic, Clinical Hospital “Sestre milosrdnice,” Zagreb, Croatia; 4 Private Gynaecological Clinic, Zagreb, Croatia; Institut national de la santé et de la recherche médicale, FRANCE

## Abstract

Change in the host and/or human papillomavirus (HPV) DNA methylation profile is probably one of the main factors responsible for the malignant progression of cervical lesions to cancer. To investigate those changes we studied 173 cervical samples with different grades of cervical lesion, from normal to cervical cancer. The methylation status of nine cellular gene promoters, *CCNA1*, *CDH1*, *C13ORF18*, *DAPK1*, *HIC1*, *RARβ2*, *hTERT1*, *hTERT2* and *TWIST1*, was investigated by Methylation Specific Polymerase Chain Reaction (MSP). The methylation of HPV18 L1-gene was also investigated by MSP, while the methylated cytosines within four regions, L1, 5’LCR, enhancer, and promoter of the HPV16 genome covering 19 CpG sites were evaluated by bisulfite sequencing. Statistically significant methylation biomarkers distinguishing between cervical precursor lesions from normal cervix were primarily *C13ORF18 *and secondly *CCNA1*, and those distinguishing cervical cancer from normal or cervical precursor lesions were *CCNA1*, *C13ORF18*, *hTERT1*, *hTERT2* and *TWIST1*. In addition, the methylation analysis of individual CpG sites of the HPV16 genome in different sample groups, notably the 7455 and 7694 sites, proved to be more important than the overall methylation frequency. The majority of HPV18 positive samples contained both methylated and unmethylated L1 gene, and samples with L1-gene methylated forms alone had better prognosis when correlated with the host cell gene promoters’ methylation profiles. In conclusion, both cellular and viral methylation biomarkers should be used for monitoring cervical lesion progression to prevent invasive cervical cancer.

## Introduction

The relationship between human papillomavirus (HPV) status and cervical cancer (CC) development is well established. There are at least 15 oncogenic or high-risk (HR)-HPV types, of which types 16 and 18 contribute up to 71% of cancer cases [1]. In contrast, DNA methylation of cellular and viral genes, resulting in silencing of gene expression, has been less studied in cervical cancerogenesis [2], even though it is one of the early events of carcinogenesis in many other cancer types [3]. DNA methylation often occurs on cytosines in CpG dinucleotides of genes for tumor suppressors, transcription factors and cell cycle regulators thus inhibiting gene expression by promoter silencing [4].

The CC occurrence is relatively rare compared with the widespread HPV infection; most women are probably infected with at least one if not several types of HPV during their sexual life, and only a small proportion will develop cervical precancerous lesions and then invasive cancer [5,6]. Factors associated with progression from precancerous cervical lesions, *i*.*e*. subclinical HPV infection to cancer are not well established. Both viral and host cells genes can be targeted by the DNA methylation machinery [4]. The fact that methylation of cellular genes can be detected even in samples taken up to seven years prior to cervical cancer diagnosis [7], was one of the reasons for this study. In addition, based on our observation the methyl groups are stable for years after DNA isolation and storage at -20°C and in line with the diagnosis taken upon sampling. Furthermore, the methylation of HPV genome could be a host defense mechanism for suppressing transcription of foreign DNA or strategy that the virus uses to maintain a long-term infection by immunosurveilance escape, or both [8,9]. We hypothesized that methylation of CpG sites in HPV16 and HPV18 genomes that represses transcription of HPV genes is one of the factors that are possibly relevant in cervical carcinogenic progression. Therefore, in this study, we focused on HPV16 and HPV18 positive cervical samples and analyzed the CpG methylation of HPV16 and HPV18 genomes as well as several key host cells genes.

There is a strong need to establish accurate diagnostic and prognostic methylation profile for a panel of genes that could discriminate between normal cervices, cervical lesions and cancer. After reviewing the literature focusing on this matter using similar sample pool and laboratory equipment [10–14], we have narrowed the choice to nine cellular genes to be investigated in this study: *CCNA1* (CCNA1, cyclin A1), *CDH1* (CDH1, cadherin 1, type 1, E-cadherin), *C13ORF18* (KIAA0226L, KIAA0226-like), *DAPK1* (DAPK1, death-associated protein kinase 1), *HIC1* (HIC1, hypermethylated in cancer 1), *RARβ2* (RARB, retinoic acid receptor, beta), *hTERT1*, *hTERT2* (TERT, telomerase reverse transcriptase), and *TWIST1* (TWIST1, twist family bHLH transcription factor 1). Promoter methylation of *CCNA1*, *C13ORF18* and *RARβ2* leads to disruption of cell cycle, methylation of *CDH1*, *DAPK1*, *hTERT1*, *hTERT2* and *HIC1* contribute to cancer progression by increasing proliferation, invasion, and/or metastasis while methylated *TWIST1* means impaired cellular differentiation. The aim of this study was to determine the methylation profile of the host cell gene promoters and simultaneously the methylation status of HPV16 and 18 genomes to identify the best methylation diagnostic and prognostic biomarkers for cervical changes.

## Results and Discussion

This is one of the few studies on a comprehensive collection number of cervical samples with well defined different cytological/histopathological diagnosis that analyzes DNA methylation of several cellular gene promoters as well as HPV16 and HPV18 genomes. Similar studies have been conducted by several authors but either on a limited number of cellular gene promoters or restricted number of analyzed HPV types [15–17].

There are some relevant literature data about methylation status of cellular genes, the following *CCNA1*, *CDH1*, *C13ORF18*, *DAPK1*, *HIC1*, *RARβ2*, *hTERT1*, *hTERT2* and *TWIST1* in cervical cancer cell lines CaSki, SiHa and HeLa [12,18–20]. Herein, methylation profiles of the nine gene promoters tested on cell lines CaSki and SiHa confirmed that all tested gene promoters were methylated in CC. In the cell line HeLa, only *DAPK1* promoter was not methylated. Methylation profiles of aforementioned gene promoters were evaluated in different sample groups: samples with normal, LSIL/CIN1, HSIL/CIN2 and HSIL/CIN3 cytology, and histopathologically confirmed CC (Table 1). In the sample group with normal cytology there were 20.0% (8/40) HPV positive with one or more HR-HPVs, of which four HPV16. The majority of the samples were methylated in most tested gene promoters. *CDH1* was found to be methylated in most cases (82.5%), followed by *HIC1* (67.5%), *RARβ2* (62.5%), and *DAPK1* (55.0%). Since they have been found highly methylated in the normal cervix, these gene promoters are not ideal methylation biomarkers for cervical carcinogenesis. Contrary to our findings, Flatley *et al*. [21] found *CDH1* promoter methylated in only 2.3% normal cervical samples. In contrast, *hTERT1* was found unmethylated in all cases. The *hTERT1* promoter seems to be a promising methylation biomarker being unmethylated in normal cervix. As far as this, there are contradictory findings of Iliopoulos *et al*. [22] who found the *hTERT1* promoter methylated in a higher proportion 26.6% in normal cervix. The fact that the methylation profile of the *hTERT1* promoter remains relatively low in cervical precursor changes, namely LSIL/CIN1 (12.5%), HSIL/CIN2 (5.1%) and HSIL/CIN3 (7.1%), and increases in CC samples (70.0%) favours our hypothesis (Table 1). Although, slightly less expressed similar methylation dynamics is observed with the *hTERT2* and *TWIST1* promoters. Accordingly, hypermethylation of all three gene promoters, *hTERT1*, *hTERT2* and *TWIST1* could be good cervical cancer biomarker, upon confirmation on larger sample pool. In the sample group with LSIL/CIN1 diagnosis there were 55.0% (22/40) HPV positive samples including six HPV16 and nine HPV18. The *CDH1* and *HIC1* promoters were methylated in the highest proportion of cases (75.0%, both), while *TWIST1* was unmethylated in all cases. In contrast, Flatley *et al*. [21] have found *CDH1* and *HIC1* promoters methylated in much lower extent in samples with the same diagnosis, in 1.8% and 7.7% cases, respectively. In line with our finding, high *HIC1* methylation (67.6%) and completely lack of methylation in *TWIST1* promoter within the same diagnosis was found by Feng *et al*. [23]. HPV was detected in 32 of 40 samples (80.0%) with HSIL/CIN2 diagnosis, including six HPV16 and seven HPV18. Because of unsuccessful bisulfite conversion in one sample, the study group further included 39 HSIL/CIN2 samples, which are specifically classified as such by the Croatian cervical cytology classification, “Zagreb 2002” [24], in order to decipher fine differences between LSIL and HSIL. Here again, the majority of samples were methylated in most tested gene promoters, *CDH1* being methylated in most cases (79.5%), while *hTERT2* was unmethylated in all cases. Among 42 samples with HSIL/CIN3 diagnosis HPV was detected in 36 samples (85.7%), including 17 HPV16 and five HPV18. The highest number of methylated cases was observed in the *CDH1* (76.2%), and the lowest in the *hTERT2* (2.4%) promoter. Other authors reported methylation of the same genes within the same sample group but slightly different percentages of methylation per each gene promoter [21,23]. However, regarding the dynamic of the methylation profile of these two gene promoters, it seems that *CDH1* promoter is constantly hypermethylated, while *hTERT2* promoter is much less methylated from normal to high grade cervical lesions. In the CC group there were 81.8% (9/11) samples positive for HR-HPV, of which seven HPV16 and one HPV18. One CC sample, stage IV-A, highly necrotic, HPV-negative and unmethylated in either gene promoter was excluded from further analysis. Most of the investigated gene promoters were methylated in this sample group, ranging from 60.0% for *DAPK1* to 100.0% for *CDH1* promoter. Among CC diagnosis *hTERT2* promoter was methylated in 60.0% of samples. In the case of *hTERT* higher gene expression is achieved by hypermethylation. In the study of Kumari *et al*. [25] treatment with 5-azacytidine and consequent demethylation of *hTERT* promoter led to reduction in gene expression. That was in contrast of what has been observed for conventional tumor suppressor genes. The possible explanation of this phenomenon is that methylation of *hTERT* promoters is heterogeneous and that only unmethylated alleles are expressed [12]. Indeed, that was observed in the study of Zinn *et al*. [26] where most of the cancer cell lines had *hTERT* promoter densely methylated, but CpGs around the transcription start site of a substantial number of *hTERT* alleles were unmethylated. In conclusion, herein observed hypomethylation in normal cervix means lower expression of *hTERT* gene and lower telomerase activity. This can explain better prognosis of patients with CC whose tumor cells lack *hTERT* promoter methylation [27]. The promoter methylation findings of other authors on CC samples correlate well with ours. The methylation varies over 65% for *hTERT* and *DAPK* promoters [22], 41% for *DAPK*, and less for *CDH1*, *HIC* and *RARβ* promoters [21]. For a higher number of promoters, *hTERT*, *CDH1*, *DAPK*, *HIC1* and *RARβ* the methylation varies even more, ranging from 0 to 100% [27–29].

**Table 1:** Methylation of the host cells gene promoters in the study group (N = 171).

Gene promoter	Number (%) of methylated samples in				
	Normal cytology^a^	LSIL/CIN1^b^	HSIL/CIN2^c^	HSIL/CIN3^d^	CC^e^
***CCNA1***	5 (12.5)	12 (30.0)	9 (23.1)	16 (38.1)	8 (80.0)
***CDH1***	33 (82.5)	30 (75.0)	31 (79.5)	32 (76.2)	10 (100.0)
***C13ORF18***	3 (7.5)	11 (27.5)	10 (25.6)	9 (21.4)	9 (90.0)
***DAPK1***	22 (55.0)	20 (50.0)	18 (46.2)	16 (38.1)	6 (60.0)
***HIC1***	27 (67.5)	30 (75.0)	27 (69.2)	24 (57.1)	8 (80.0)
***RARβ2***	25 (62.5)	14 (35.0)	24 (61.5)	26 (61.9)	9 (90.0)
***hTERT1***	0 (0.0)	5 (12.5)	2 (5.1)	3 (7.1)	7 (70.0)
***hTERT2***	3 (7.5)	5 (12.5)	0 (0.0)	1 (2.4)	6 (60.0)
***TWIST1***	11 (27.5)	0 (0.0)	9 (23.1)	9 (21.4)	8 (80.0)

^a^N = 40;

^b^N = 40;

^c^N = 39;

^d^N = 42;

^e^N = 10;

LSIL, low-grade squamous cell intraepithelial lesion; HSIL, high-grade squamous cell intraepithelial lesion; CIN, cervical intraepithelial neoplasia; CC, cervical cancer.

**Fig 1:**
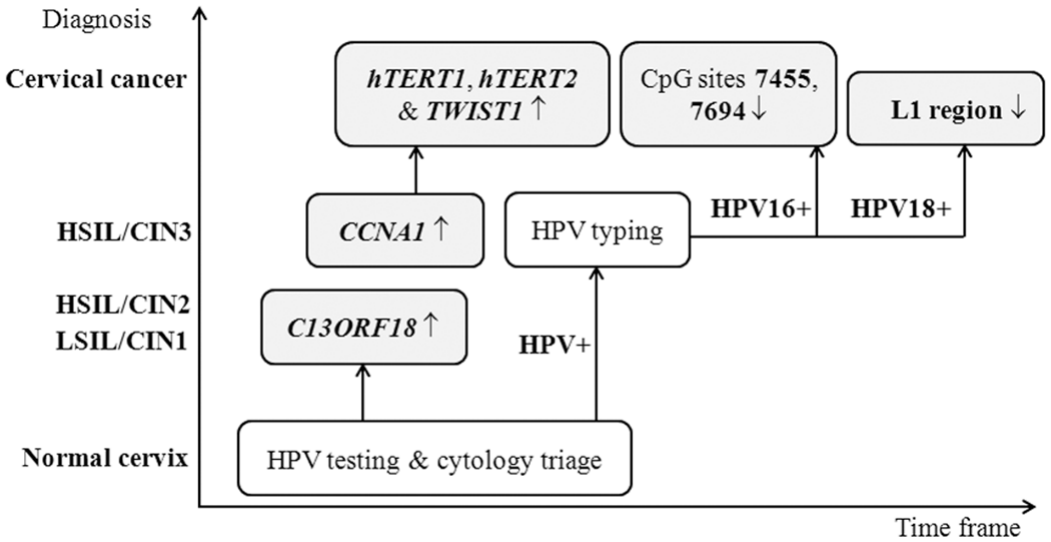
Suggested algorithm for DNA methylation potential biomarkers use in clinical practice. LSIL, low-grade squamous cell intraepithelial lesion; HSIL, high-grade squamous cell intraepithelial lesion; CIN, cervical intraepithelial neoplasia; ↑, hypermethylated; ↓, hypomethylated.

Herein, we have identified five main methylation biomarker candidates, *CCNA1*, *C13ORF18*, *hTERT1*, *hTERT2*, and *TWIST1*, whose promoter methylation status distinguishes CC from normal and HSIL/CIN3 samples (Table 2). The progression from HSIL/CIN1 to HSIL/CIN2 seems to be related to higher methylation of *RARβ2* and *TWIST1* promoter, although the statistical significance is lower for *RARβ2* (Fisher’s exact test p = 0.024696) than *TWIST1* (Fisher’s exact test p = 0.001030). The late stage of cervical changes, CC seems to be characterized by high methylation of *hTERT1*, *hTERT2*, and *TWIST1* promoter, and in a lower extent in *RARβ2* promoter. We also grouped cervical precursor lesions (LSIL/CIN1, HSIL/CIN2 and HSIL/CIN3) to evaluate differences between normal, cervical lesions, and CC. In such comparison *CCNA1* and *C13ORF18* promoters revealed to be statistically significantly hypermethylated in cervical precursor lesions compared to normal cervices (Fisher’s exact test p = 0.023742 and 0.022603, respectively). In addition, the methylation of these two potential biomarkers compared to normal cervices seems to be an early event in cervical cancerogenesis, with *C13ORF18* promoter being statistically significantly methylated in LSIL/CIN1 and HSIL/CIN2 (Fisher’s exact test p = 0.036738 in both cases), while *CCNA1* promoter being statistically significantly methylated in HSIL/CIN3 (Fisher’s exact test p = 0.010994). Therefore, statistically significant methylation differences between normal cervices, lesions and CC have been observed for five gene promoters: *CCNA1*, *C13ORF18*, *hTERT1*, *hTERT2* and *TWIST*. In summary, those gene promoters represent possible good methylation biomarkers for detection of cervical changes and could be useful in clinical practice. It seems that in cervical cancerogenesis there is a cascade of DNA methylation. Primarily some gene promoters are methylated, such as *C13ORF18* followed by *CCNA1*, and then others, namely *hTERT1*, *hTERT2* and *TWIST1* (Fig 1).

**Table 2:** Comparison of methylation status of gene promoter in cervical samples with different diagnosis—only statistically significant (Fisher’s exact test) increased methylation changes are presented.

Hypermethylation (N)	Gene promoter	p
**LSIL/CIN1 (40) *vs*. normal (40)**	*C13ORF18*	0.036738
**HSIL/CIN2 (39) *vs*. normal (40)**	*C13ORF18*	0.036738
**HSIL/CIN3 (42) *vs*. normal (40)**	*CCNA1*	0.010994
**HSIL/CIN2 (39) *vs*. LSIL/CIN1 (40)**	*RARβ2*	0.024696
	*TWIST1*	0.001030
**HSIL (81) *vs*. normal (40)**	*CCNA1*	0.042593
	*C13ORF18*	0.043737
**LSIL + HSIL (121) *vs*. normal (40)**	*CCNA1*	0.023742
	*C13ORF18*	0.022603
**CC (10) *vs*. normal (40)**	*CCNA1*	0.000086
	*C13ORF18*	0.000001
	*hTERT1*	0.000001
	*hTERT2*	0.000866
	*TWIST1*	0.003709
**CC (10) *vs*. HSIL/CIN3 (42)**	*CCNA1*	0.031319
	*C13ORF18*	0.000107
	*hTERT1*	0.000090
	*hTERT2*	0.000067
	*TWIST1*	0.000969
**CC (10) *vs*. LSIL + HSIL (121)**	*CCNA1*	0.003007
	*C13ORF18*	0.000070
	*RARβ2*	0.042019
	*hTERT1*	0.000017
	*hTERT2*	0.000026
	*TWIST1*	0.000031

LSIL, low-grade squamous cell intraepithelial lesion; HSIL, high-grade squamous cell intraepithelial lesion; CIN, cervical intraepithelial neoplasia; CC, cervical cancer.

Other authors determined some of the selected genes as good biomarkers, but mostly as a single biomarker to distinguish a particular diagnosis. For instance, Yang *et al*. [13] selected *CCNA1* and *C13ORF18* as good methylation biomarkers to distinguish samples with CIN2+ diagnosis from normal samples. Kitkumthorn *et al*. [18] chose hypermethylation of *CCNA1* promoter as biomarker for early diagnosis of invasive CC, De Wilde *et al*. [12] chose differentiated methylation of *hTERT* to diagnose CC, while Eijsink *et al*. [14] identified *C13ORF18* and *TERT*, together with *EPB41L3* and *JAM3*, as best methylation biomarkers for CC. Therefore, from the literature it is difficult to determine best methylation biomarkers. In a genome-wide study with Illumina Infinium HumanMethylation450 BeadChip method we confirmed the high methylation level of *CCNA1*, *C13ORF18*, *hTERT1*, *hTERT2* and *TWIST1* promoters in CC compared with the normal tissue. However, the unsupervised hierarchical clustering led to another set of genes as candidate biomarkers, *RGS7*, *LHX8*, *STGALNAC5*, *TBX20*, *KCNA3*, and *ZSCAN18* [30], which were not evaluated herein. Furthermore, in the same study [30], using the mRNA expression data sets for external validation, we demonstrated a good coherence between DNA methylation data and the gene expression array. Therefore, we can conclude that DNA methylation is a good predictor of gene expression; consequently, DNA methylation tests are good and sufficient choice to improve diagnostics, staging and prognostics in clinical investigation because of the cost and time benefit over gene expression analysis.

**Fig 2:**
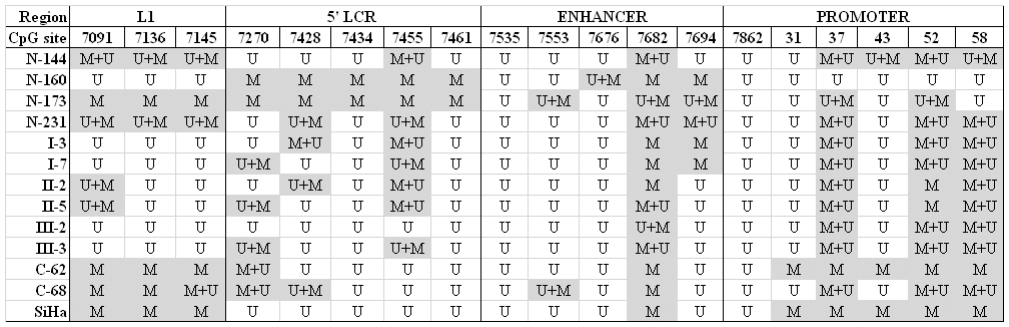
HPV16 methylation status of 19 CpG sites in different regions of the viral genome. N, normal cytology; I, LSIL/CIN1 diagnosis; II, LSIL/CIN2 diagnosis; III, HSIL/CIN3 diagnosis; CC, cervical cancer; SiHa, cervical cancer cell line; M, methylated; U, unmethylated; M+U, methylated and unmethylated with predominantly methylated DNA; U+M, methylated and unmethylated with predominantly unmethylated DNA.

**Fig 3:**
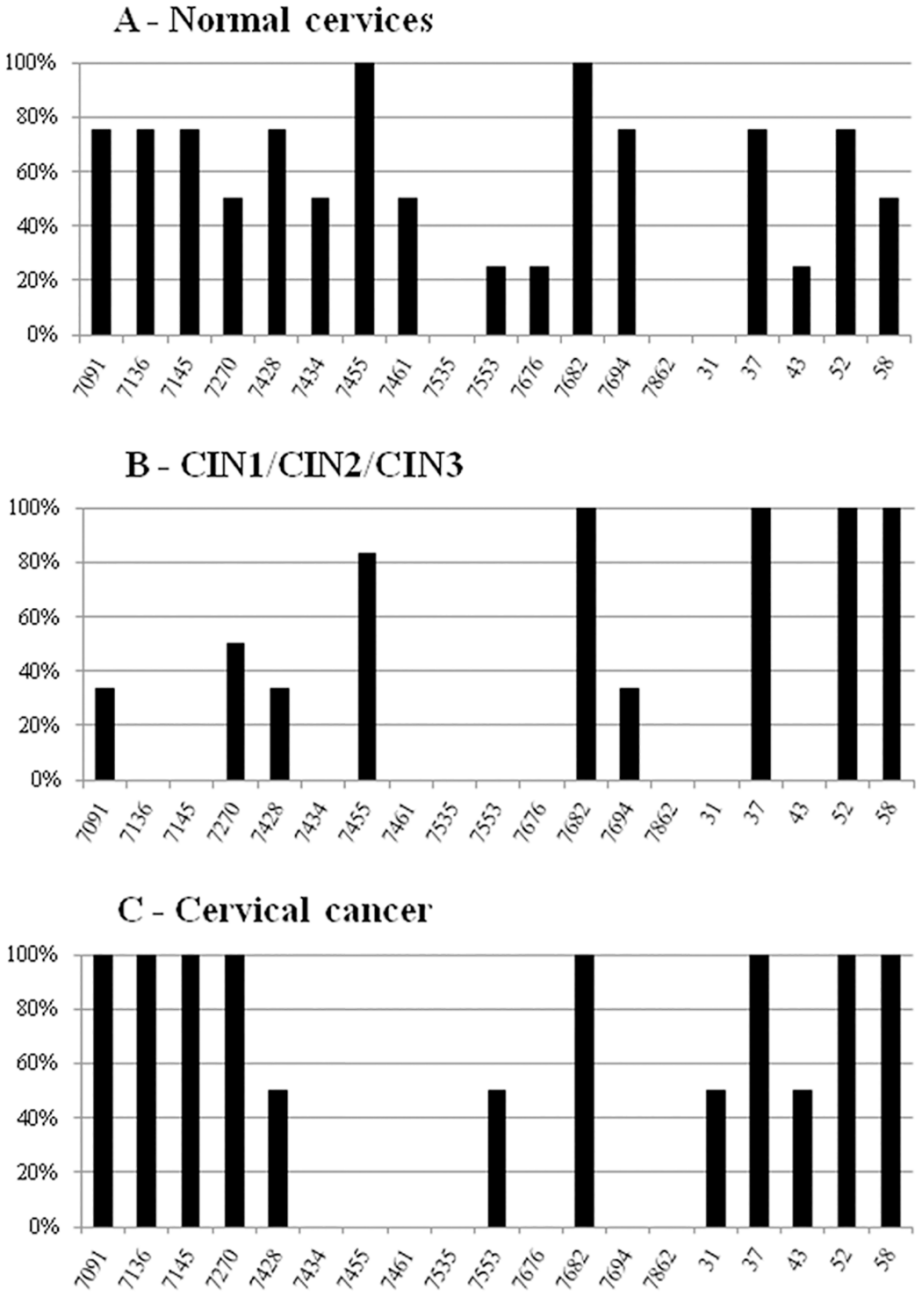
Percentage of methylation of 19 CpG sites in HPV16 genome. Normal cervices (N = 40); cervical precursor lesions, LSIL/CIN1, HSIL/CIN2 and HSIL/CIN3 (N = 121); cervical carcinoma samples (N = 10). CpG sites that were methylated and unmethylated in the same sample are presented herein as methylated.

We also investigated the CpG methylation status of HPV16 and HPV18 genomes in correlation with cytological/histopathological diagnosis and the methylation status of cellular genes. The methylation patterns of HPV16 genome has been analyzed by bisulfite sequencing for each of 19 CpG sites due to discrepant literature data. As expected, the methylation patterns of HPV16 genome in 12 samples with different diagnoses, correlated with those of the SiHa cell line (S1 Table). Most methylated CpGs in HPV16 genome of SiHa cells were detected in L1 and promoter region, while most of unmethylated CpGs in 5’LCR region and enhancer (Fig 2). Often both copies of HPV16, methylated and unmethylated were found in all diagnosis ranging from normal cervices to cervical cancer, in which the methylation profiles of CC samples were very similar to one of SiHa cell line. In almost all samples with normal cytology (N) the methylation profile was different from those in SiHa cells and CC samples. All three groups of samples with cervical precursor changes (LSIL/CIN1, HSIL/CIN2 and HSIL/CIN3) had very similar HPV16 methylation profiles. Because of this similarity of the methylation pattern, all three cervical precursor changes are shown together on Fig 3B. In addition, all CpG sites that were methylated and unmethylated in the same sample are presented as methylated (Fig 3). Our results support findings of Kalantari *et al*. [31] that HPV genome is normally hypermethylated and needs only one copy to be transcriptionally active. Moreover, as presented herein, for a good methylation biomarker it is more important to accurately determine the methylation percentage of specific CpG sites within HPV16 genome than the overall methylation in different sample groups. Herein, we observed that the cervical precancerous lesion group had the lowest overall methylation rate (33%), while samples with normal cytology and CC had 53% overall methylation rate, both. These results are partially in disagreement with the conclusions of other authors who claim that methylation of HPV16 is the lowest in normal and the highest in CC samples [17,32,33]. In this study, from the pool of 19 examined CpG sites of HPV16 genome, two seem to be the most significant, CpG 7455 (5’LCR) and CpG 7694 (enhancer), in which the complete lack of methylation was found in carcinoma samples and SiHa cell line, while they were highly methylated (100% of normal cervices and 83% of precursor lesions in CpG 7455) and moderately methylated (75% of normal cervices and 33% of precursor lesions in CpG 7694) in other sample groups. Further, CpGs at positions 7434 and 7461 were methylated in 50% of samples with normal cytology but no methylation was observed in precursor lesions, CC or SiHa cell line. In contrast, CpG 31 was methylated only in CC samples (50%) and SiHa cell line. CpGs at positions 37 and 52 were methylated in 75% samples with normal cytology and 100% of precursor lesions, CC and SiHa cell line. CpG 58 was methylated in 50% samples with normal cytology and 100% of precursor lesions and CC, as well as in SiHa cell line. The methylation was not observed in CpGs at positions 7535 and 7862 in neither one of the samples or SiHa cell line. In contrast, CpG 7682 was methylated in all examined samples, precursor cervical lesions, CC and SiHa cell line (Figs 2 and 3). Different methylation frequencies within HPV16 genome on the same CpG sites from our findings and different conclusions have been reported by Kalantari *et al*. [34]. However, similar findings on CpG methylation profiles in CC samples to ours were identified in the study of Bhattacharjee and Sengupta [35]. Thus, the complete lack or low CpG methylation in 5’LCR and enhancer, rather than in promoter region is crucial for HPV activity and consequently immortalization of host cells.

Investigating HPV18 L1-gene methylation on 22 samples (S1 Table) by MSP method we observed that it correlates with the cytological/histopathological diagnosis, and can better predict disease prognosis. Particularly, most of the analyzed samples and HeLa cell line contained both methylated and unmethylated forms of HPV18 L1-gene (Table 3). The fact is that the L1-gene remains intact in cervical lesions and cancer samples, even when HPV genome integrates into a host cell [36]. Methylation of HPV18 L1-gene in cervical precursor lesions and CC samples was in line with the diagnosis and the methylation profile of HeLa cell line. Particularly, our samples containing only methylated form of HPV18 L1-gene had better prognosis; *i*.*e*. in those samples cellular genes were methylated at a lower extent and mostly those that have not been statistically proven as potential biomarkers. Turan *et al*. [37,38] also found both copies of HPV18 in HeLa cell line, with the excess of methylated form, while hypermethylation of HPV18 L1 in all carcinomas and lack of methylation in the majority of asymptomatic smears and low-grade lesions. Furthermore, Badal *et al*. [39] have declared that HPV18 genome hypermethylation in HeLa cell line and CC samples indicate that HPV is indeed targeted by cellular DNA methylation machinery that may affect late and early gene transcription.

**Table 3:** HPV18 L1-gene methylation status HPV18 in 22 samples with mild, moderate and high grade dysplasia, and cervical cancer.

Diagnosis (N)	Number of samples (%)		
	only methylated	both methylated and unmethylated	only unmethylated
**LSIL/CIN1 (9)**	2 (22.2)	7 (77.8)	0 (0.0)
**HSIL/CIN2 (6)**	1 (16.7)	5 (83.3)	0 (0.0)
**HSIL/CIN3 (6)**	3 (50.0)	2 (33.3)	1 (16.7)
**CC (1)**	0 (0.0)	1 (100.0)	0 (0.0)

LSIL, low-grade squamous cell intraepithelial lesion; HSIL, high-grade squamous cell intraepithelial lesion; CIN, cervical intraepithelial neoplasia; CC, cervical cancer.

Future studies should validate the use of methylated HR-HPV as a predictive and/or diagnostic biomarker for risk of cervical cancer among HPV-positive women [32,40,41]. Particularly, MSP primers for HPV16 genome that cover CpGs 7455 and 7694 should be designed in order to avoid expensive and time consuming bisulfite sequencing, and, as such, simplify clinical applications. Our consideration of HPV genome methylation testing together with the cellular gene methylation testing as triage of women at high-risk for developing cervical cancer was also already discussed [10,27,42], but the question remains which HPV types, which HPV genome regions and which cellular genes to analyze. We believe that the results presented in this study, implied after confirmation on a larger number of samples, could resolve those concerns. Therefore, we would suggest that all HR-HPV-positive women get tested for methylation of the *CCNA1*, *C13ORF18*, *hTERT1*, *hTERT2* and *TWIST1* promoters as well as at least HPV16 CpG 7455 and 7694 sites, and HPV18 L1-gene methylation. The test results should be correlated, and in case of increased methylation of candidate biomarker genes and changed methylation profile of HPV16 and 18 genome women should be managed and subsequently monitored more thoroughly (Fig 1). In addition, based on actual knowledge on cellular and viral DNA methylation the possible use of demethylation drugs [27,43], such as DNA methyltransferases inhibitors could be considered as therapeutics but should be applied with great caution.

## Material and Methods

### Study group

The study group included 173 cervical specimens of women with normal cytology, precancerous lesions (squamous cell intraepithelial lesions, low-grade, LSIL and high-grade, HSIL), *i*.*e*. cervical intraepithelial neoplasia (CIN) grade 1 to 3, classified according to “Zagreb 2002”, that is in line with “NCI Bethesda System 2001” [24], and histopathologically confirmed CC [44]. From the pool of samples collected for regular diagnostics at the Rudjer Boskovic Institute, Zagreb, 40 samples with normal cytology, 40 with LSIL/CIN1, 40 with HSIL/CIN2 and 42 samples with HSIL/CIN3 diagnosis were taken randomly for methylation analyses. In addition, 11 CC (8 squamous cell carcinoma and 3 adenocarcinoma) samples were taken with cytobrush just before the surgical procedures.

### Ethics Statement

Verbal patient/participant consent was obtained for each cervical specimen that was collected both for HPV diagnostic and research purposes. Written patient/participant consent was not necessary because each cervical sample is accompanied by the Laboratory service request forms, which have to be signed and approved by the practicing physician who is responsible for the verbal patient/participant consent (recorded by the clinician on the Laboratory service request forms). Thus, only samples of patients/participants who verbally agreed were included in this study. The relevant patient data (age, cytological diagnosis, HPV detection and typing result) and the extracted DNA were further encoded and processed anonymously in the laboratory. The study was achieved within the research project “Aberrant DNA methylation in HPV associated lesions” (Grant 098-0982464-2510 supported by the by the Croatian Ministry of Science, Education and Sport), which was approved as well as the sample collection procedure by the Ethical Board of the Rudjer Boskovic Institute, and the Ethical Board of Sisters of Mercy Hospital, and Clinical Hospital Center Zagreb that is in line with the Helsinki declaration (DoH/Oct2008).

### DNA preparation

DNA from cervical samples was isolated on the BioRobot EZ1 (Qiagen, USA) according to the manufacturer’s instruction. After DNA extraction, the purified DNA was dissolved in 50–100 μl of tri-distillate sterile water and stored at -20°C until further analysis. Each DNA was analyzed spectrophotometrically and visually on 1% agarose gel electrophoresis, and visualized by UV irradiation on Alliance 4.7 (UVItec Cambridge, UK) [45].

### HPV detection and genotyping

Detection and genotyping of HPVs was previously described by Milutin-Gasperov *et al*. [46] Briefly, three sets of consensus primers (PGMY09/PGMY11, L1C1/L1C2-1/L1C2-2 and GP5+/GP6+) were used for HPV detection, and type-specific primers for HPV genotyping of types 6/11, 16, 18, 31, 33, 45, 52 and 58. PCR products were analyzed by electrophoresis on 2% agarose gels stained with ethidium bromide.

### Cell lines

Isolated DNAs from HPV16 positive CC cell lines, CaSki (ATTC CRL-1550) and SiHa (ATCC HTB-35), and HPV18 positive, HeLa (ATTC CCL-2), were used as positive controls of aberrant methylation profiles of cellular genes and respective HPV genomes. CaSki cells contain an integrated HPV16 genome (about 600 copies per cell) as well as sequences related to HPV18. SiHa cells contain an integrated HPV16 genome (1 to 2 copies per cell), while HeLa cells contain HPV18 sequences.

### Bisulfite DNA conversion

DNA of cervical samples was modified with sodium bisulfite using EpiTect Bisulfite Kit (Qiagen, USA) and then purified according to manufacturer’s instructions. Briefly, all unmethylated cytosines are converted to uracil by bisulfite treatment, while methylated cytosines remain unchanged.

### Host cell gene promoters’ methylation determination

The methylation profiles of *CCNA1* (Chromosome 13: 36432177–3643232), *CDH1* (Chromosome 16: 68737114–6873722), *C13ORF18* (Chromosome 13: 46387345–4638746), *DAPK1* (Chromosome 9: 87497883–87497981), *HIC1* Chromosome 7: 19117831–19118031, *RARβ2* (Chromosome 3: 25428191–2542842), *hTERT1* (Chromosome 5: 1295019–1295259), *hTERT2* (Chromosome 5: 1294824–1295014) and *TWIST1* (Chromosome 7: 19117831–19118031) gene promoters were identified. Gene promoter methylation status was obtained by Methylation Specific Polymerase Chain Reaction (MSP) with primers specific for methylated forms of gene promoters. The same method was used for detection of unmethylated forms of the same gene promoters except for the *C13ORF18* promoter. The positivity of samples with unmethylated forms of gene promoters served as the internal control, since cervical smears contain mixtures of cells with diverse methylation status of the host gene promoters [17]. The MSP method and conditions for the selected genes were already described by Yang *et al*. [13,47], Feng *et al*. [23], Kitkumthorn *et al*. [18], and Dessain *et al*. [19], but the conditions were modified in most cases herein. Briefly, 5 min denaturation at 95°C was followed by 45 cycles of denaturation at 95°C for 30s, annealing at 56–61°C for 30s, elongation at 72°C for 50s, and the final 7 min extension at 72°C. The annealing temperature varied with different primers: 56°C for *hTERT1* (U, unmethylated primers) and *TWIST1* (M, methylated primers), 58°C for *CCNA1* (M) and *CCNA1* (U), 59°C for *CDH1* (U), *C13ORF18* (M), *DAPK1* (U), *HIC1* (U), *RARβ2* (M) and *TWIST1* (U), and 60°C for *DAPK1* (M), *HIC1* (M), *RARβ2* (U), *hTERT1* (M), *hTERT2* (M) and *hTERT2* (U) and 61°C for *CDH1* (M). MSP was carried out for each primer-set separately in a 20 μl volume containing 0.4 mM each dNTP, 1 μM each primer, 4 mM MgCl_2_, 5x Green GoTaq Flexi buffer, H_2_O and 0.08 μl GoTaq Hot Start Polymerase (Promega, USA). Bisulfite converted specimens’ DNA or methylated/unmethylated control DNA (EpiTect Control DNA, methylated and unmethylated, Qiagen, USA) were added into reactions in the final concentrations of 25 ng/μl. Aliquots of MSP products were analyzed on a 2% agarose gel.

### HPV16 genome methylation determination

Bisulfite sequencing was used to identify the number and the position of the methylated cytosines inside four regions (L1, 5’LCR, enhancer, and promoter) of the HPV16 genome covering 19 CpG sites [34]. The PCR reactions were performed in a 50 μl volume containing 1 mM each dNTP, 2.5 μM each primer, 10 mM MgCl_2_, 5x Green GoTaq Flexi buffer, H_2_O and 0.2 μl GoTaq Hot Start Polymerase. Bisulfite converted specimens’ DNA and those isolated from CaSki cell line were added into reactions in the final concentration of 25 ng/μl. The PCR conditions consisted of: 5 min denaturation at 95°C followed by 45 cycles of 30s denaturation at 95°C, 30s annealing at 50°C (16msp4F/7R primers for enhancer), 53°C (16msp3F/3R primers for L1 and 5’LCR), or 55°C (16msp5F/8R primers for promoter), and 1 min extension at 68°C with the final 7 min extension at 68°C. The amplified products were analyzed by electrophoresis on 2% agarose gels and purified with Wizard PCR and Gel Cleanup kit (Promega, USA) according to manufacturer’s instructions. Purified PCR products for bisulfite modified DNA sequencing were analyzed at the Rudjer Boskovic Institute DNA Service, Zagreb, by the ABI PRISM 3100-Avant Genetic Analyzer (Applied Biosystems, USA).

### HPV18 L1-gene methylation determination

MSP with primers specific for methylated and unmethylated forms of the L1-gene (between 7017 and 7140 positions) of HPV18 was used to amplify bisulfite converted DNA [37]. Sample or HeLa bisulfite converted DNA (positive control) in the final concentrations of 25 ng/μl was used in the MSP reaction that was carried out in a 20 μl volume containing 0.4 mM each dNTP, 1 μM each primer, 4 mM MgCl_2_, 5x Green GoTaq Flexi buffer, H_2_O and 0.08 μl GoTaq Hot Start Polymerase. MSP conditions were 5 min denaturation at 95°C, followed by 45 cycles of 95°C for 30s, 58°C for 30s and 68°C for 1 min with the final 7 min extension at 68°C. The amplified products were analyzed on a 2% agarose gels.

### Statistics

Since the number of positive samples in the sample groups was small, the Fisher's exact test was used to determine the correlation of methylation frequencies between different diagnoses. Statistically significant differences were considered those with p-value<0.05. The data were processed in a computer program MedCalc (version 11.4.2).

## Supporting Information

S1 Table Characteristics of samples used for testing methylation status of HPV16 (N = 12) and HPV18 (N = 22) genome. (DOC)
