# Supplementary material for: Methylated Host Cell Gene Promoters and Human Papillomavirus Type 16 and 18 Predicting Cervical Lesions and Cancer
Source: PLoS One. 2015 Jun 9;10(6):e0129452. doi: 10.1371/journal.pone.0129452 (PMC4461273; doi:10.1371/journal.pone.0129452)
Supplement: S1 Table — (DOC) [file pone.0129452.s001.doc]

**S1 Table. Ch**aracteristics of samples used for testing methylation status of HPV16 (N=12) and HPV18 (N=22) genome.

|  | **Diagnosis** | **Age** | **HPV type(s)** |
| --- | --- | --- | --- |
| **HPV16 positive samples** | | | |
| **N-144** | normal cytology | 40 | 16 |
| **N-160** | normal cytology | 28 | 16 |
| **N-173** | normal cytology | 22 | 16, 52 |
| **N-231** | normal cytology | 33 | 16 |
| **I-3** | LSIL/CIN1 | 21 | 16, 18, 58 |
| **I-7** | LSIL/CIN1 | 24 | 16, 18 |
| **II-1** | HSIL/CIN2 | 29 | 16 |
| **II-5** | HSIL/CIN2 | 22 | 16, 18, 52, 58 |
| **III-2** | HSIL/CIN3 | 44 | 16 |
| **III-3** | HSIL/CIN3 | 23 | 16 |
| **CC-62** | stage IIB | 61 | 16 |
| **CC-68** | stage IIIB | 63 | 16 |
| **HPV18 positive samples** | | | |
| **I-21** | LSIL/CIN1 | 32 | 18 |
| **I-69** | LSIL/CIN1 | 29 | 18 |
| **I-102** | LSIL/CIN1 | 22 | 6/11, 18 |
| **I-138** | LSIL/CIN1 | 21 | 16, 18, 58 |
| **I-189** | LSIL/CIN1 | 23 | 16, 18, 52 |
| **I-323** | LSIL/CIN1 | 23 | 18 |
| **I-376** | LSIL/CIN1 | 33 | 18 |
| **I-428** | LSIL/CIN1 | 29 | 18 |
| **I-500** | LSIL/CIN1 | 24 | 16, 18 |
| **II-88** | HSIL/CIN2 | 24 | 18, 45 |
| **II-89** | HSIL/CIN2 | 32 | 18 |
| **II-120** | HSIL/CIN2 | 21 | 18, 52, 58 |
| **II-148** | HSIL/CIN2 | 46 | 18, 31 |
| **II-273** | HSIL/CIN2 | 24 | 18 |
| **II-538** | HSIL/CIN2 | 22 | 16, 18, 52, 58 |
| **III-62** | HSIL/CIN3 | 24 | 18 |
| **III-83** | HSIL/CIN3 | 32 | 18, 31 |
| **III-133** | HSIL/CIN3 | 29 | 18 |
| **III-294** | HSIL/CIN3 | 26 | 18 |
| **III-448** | HSIL/CIN3 | 35 | 16, 18 |
| **III-482** | HSIL/CIN3 | 25 | 6/11, 18 |
| **CC-59** | stage IB1 | 35 | 18 |

LSIL, low-grade squamous cell intraepithelial lesion; HSIL, high-grade squamous cell intraepithelial lesion; CIN, cervical intraepithelial neoplasia; CC, cervical cancer.
